# Bilateral Distal Femoral Nailing in a Rare Symmetrical Periprosthetic Knee Fracture

**DOI:** 10.1155/2014/745083

**Published:** 2014-12-14

**Authors:** Marcos Carvalho, Ruben Fonseca, Pedro Simões, André Bahute, António Mendonça, Fernando Fonseca

**Affiliations:** Orthopaedics Department, Coimbra Hospital and University Center, Rua Fonseca Pinto, 3000-075 Coimbra, Portugal

## Abstract

The authors report a case of a 78-year-old polytrauma patient, with severe thoracic trauma and bilateral symmetrical periprosthetic femoral fractures after a violent car accident. After the primary survey, with the thoracic trauma stabilized, neurovascular lesions excluded, and provisional immobilization applied, both fractures were classified as OTA: 33-A3, Rorabeck Type II, and closed reduction and internal fixation with distal femoral nails were performed. At 5 months of follow-up, the patient was able to walk with crutches and clear radiologic signs of fracture consolidation could be seen. At 24 months, the patient walked without any walking aid and had recovered her previous functional status. This surgical option allowed the authors to achieve relative stability using an intramedullary technique, preserving fracture hematoma in an osteopenic patient, and was found to be successful in recovering the patient's previous functional status and satisfaction after major trauma.

## 1. Introduction

Periprosthetic femoral fractures above total knee replacement are an uncommon condition (0,3–3%) [1–3] that is becoming more frequent, in possible relation with the growing number of knee arthroplasties. This type of fractures, commonly seen in older patients, is often caused by minor trauma such as a fall from standing height and less frequently by high-energy trauma (road-traffic accidents, seizures, or forced manipulation of a stiff knee).

Risk factors for this condition include osteoporosis, rheumatoid arthritis, neurologic disorders, chronic steroid therapy, anterior cortical notching of the femur, local osteolysis, local infection, and revision knee arthroplasty [4–7].

This type of fracture requires meticulous classification and clinical evaluation based on the location and stability of the prosthetic components. This information, supported by clinical examination and imaging results, is crucial to plan the surgical approach, in order to manage the best option among the variety of implants, methods, and principles available.

Because it is possible to treat this type of fracture with different reduction techniques, stability principles, and arthroplasty options, we found it important to share our experience and results with the use of relative stability with a distal intramedullary technique, in a rare pattern fracture, a OTA: 33-A3.2 bilateral fracture. With this method of closed reduction, we were able to achieve indirect bone healing by preserving fracture hematoma with its local osteogenic stem cells, inductive proteins, and chemical mediators—what we refer to as the callus induction cocktail—and obtain good functional results at 2 years of follow-up in an osteopenic patient.

## 2. Case Presentation

**Figure 1:**
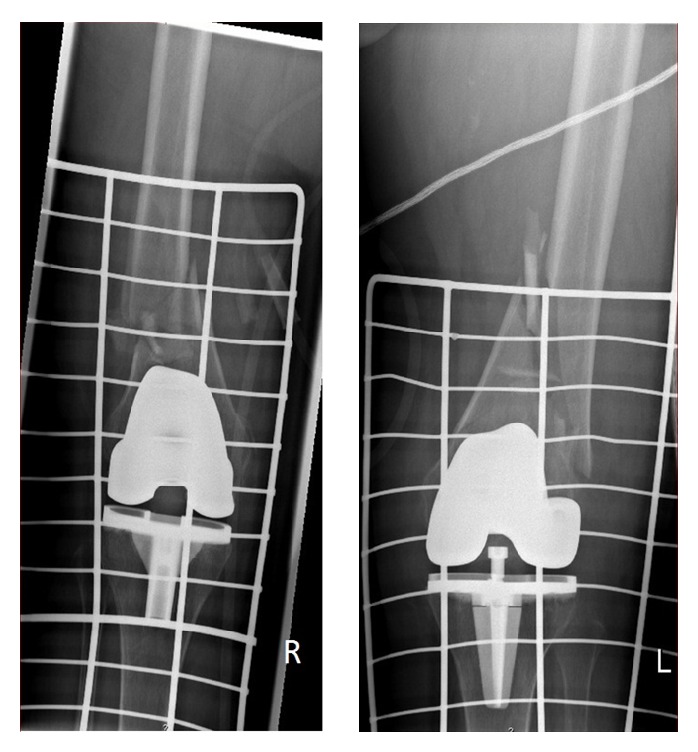
Preoperative AP X-rays showing bilateral symmetrical periprosthetic femoral fracture above knee arthroplasties.

A 78-year-old female, with history of bilateral total knee arthroplasty (TKA), presented to the emergency department after a car accident. Clinical examination revealed a flail chest, respiratory distress and limb deformity around the knees, crepitus and abnormal mobility without signs of neurovascular lesion, and a soft tissue lesion around the middle third of her right leg. Plain film radiography showed five fractured ribs on the left hemithorax and two almost identical periprosthetic femoral fractures, classified as OTA: 33-A3.2 and as a type II in Rorabeck classification of periprosthetic fractures (Figure 1).

The patient was resuscitated, intubated, and mechanically ventilated and a chest tube was implanted for drainage. Our trauma team conducted provisional alignment and splint immobilization of both lower limbs and evaluation of the neurovascular status and soft tissue viability.

**Figure 2:**
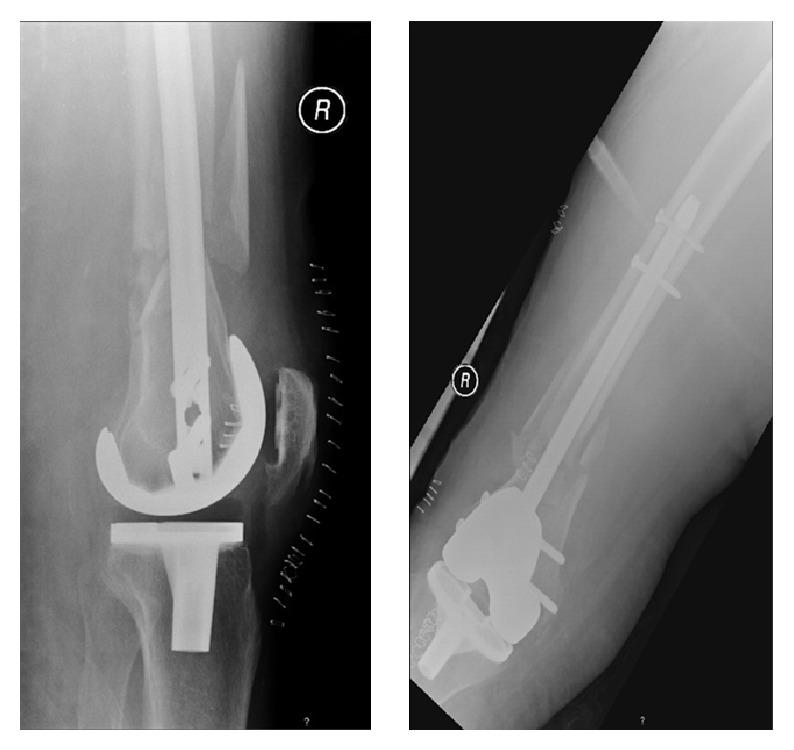
Immediate postoperative AP and lateral X-rays showing a right periprosthetic knee fracture, stabilized with retrograde intramedullary nailing technique.

**Figure 3:**
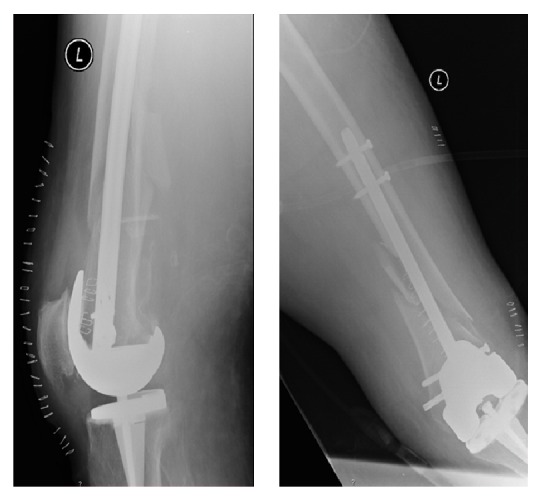
Immediate postoperative AP and lateral X-rays showing a left periprosthetic knee fracture, stabilized with retrograde intramedullary nailing technique.

Six days after admission, the patient underwent surgical closed reduction and internal fixation of both femoral fractures with distal femoral nails (Figures 2 and 3). Postoperatively, she developed pneumonia and worsening of her respiratory function, which prolonged her stay in our intensive care unit for three months. There were no surgical complications observed. The patient was discharged 4 months after the traumatic event.

**Figure 4:**
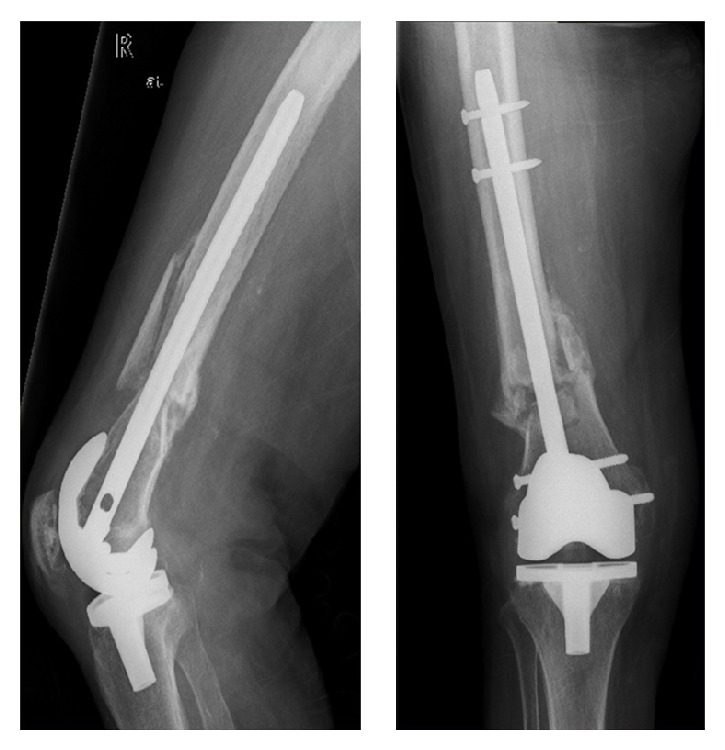
Five months of postoperative AP and lateral right knee X-rays showing signs of bone healing.

**Figure 5:**
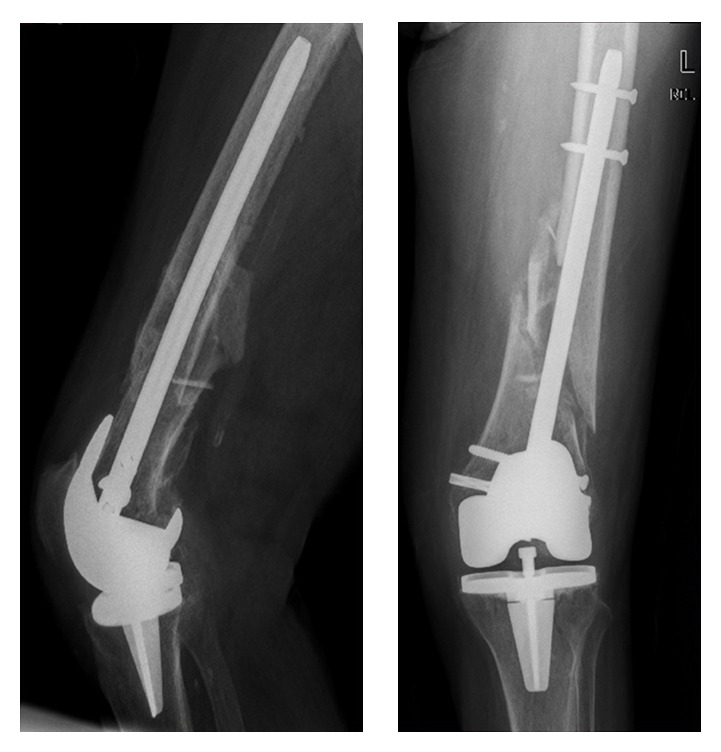
Five months of postoperative AP and lateral left knee X-rays showing signs of bone healing.

**Figure 6:**
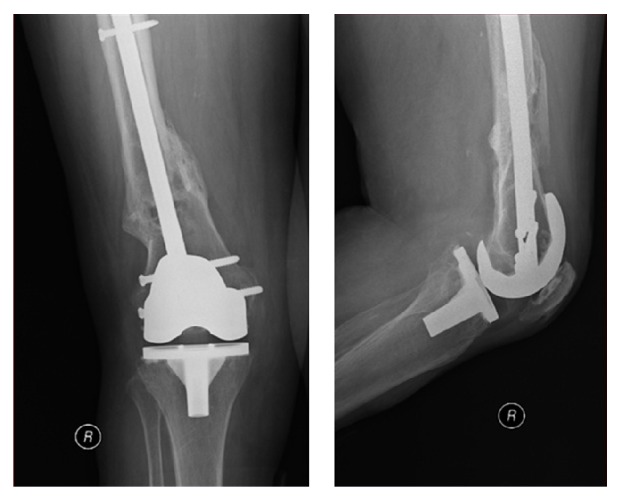
24 months of postoperative AP and lateral right knee X-rays showing complete consolidation of the fracture.

**Figure 7:**
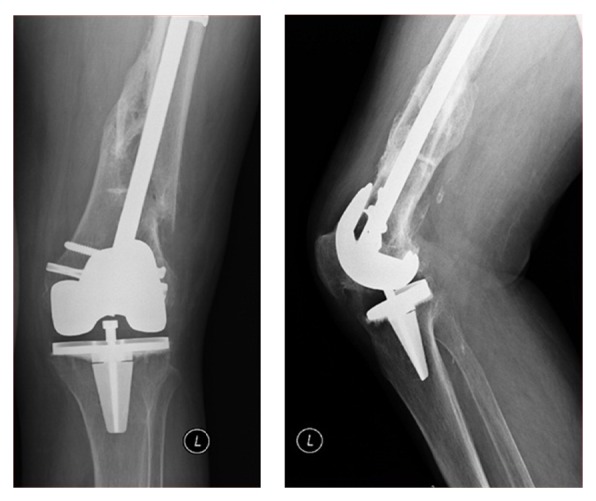
24 months of postoperative AP and lateral left knee X-rays showing complete consolidation of the fracture.

**Figure 8:**
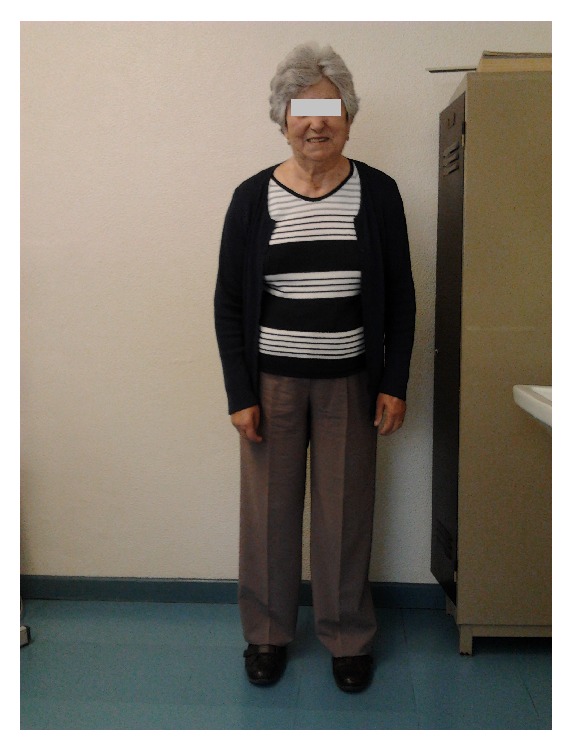
At 2 years postoperatively the patient is able to walk without any walking aids and had recovered her previous functional status.

Five months after surgery (Figures 4 and 5), the patient was able to walk with crutches and there were clear radiologic signs of fracture consolidation. At 24 months, bone consolidation was obvious (Figures 6 and 7); the patient walked without any walking aids and had recovered her previous functional status (Figure 8).

## 3. Discussion

Nowadays we are observing a growing number of TKA procedures as surgical indication is becoming more flexible, life expectancy increases, and elderly patients are becoming more active. As a consequence, the incidence of long-term complications such as periprosthetic knee fractures, currently ranging from 0,3 to 3% [1–3], is also likely to increase in the future. Most of these fractures result from axial and torsional loads and are related to low energy mechanisms. However, in 10% of the cases, they can present following high energy injuries [8].

This type of periprosthetic fractures remains a major challenge to orthopaedic surgeons, with a large variety of implants, designs, concepts, and principles needed to be considered for each patient, on an individual basis.

The purpose of the surgical procedure in this type of fractures is to preserve limb length, restore rotational alignment, and allow early motion. A variety of treatment options are described in the literature depending on the stability of the prosthetic components, fracture pattern, bone stock quality, presence of any other implants, and general physical condition of the patient [9].

In our case, the patient had symmetrical bilateral periprosthetic knee femoral fractures and osteopenic bone, which led us to select a closed reduction method with a load-sharing intramedullary device, leaving the site of fracture untouched and allowing for indirect bone healing by a relative stability method. After 2 years of follow-up, the patient was able to walk and recovered her previous functional status and satisfaction after this major traumatic event.

In planning the surgical procedure, when using a retrograde intramedullary nail, it is important to know which femoral components were used in the original arthroplasty, since the component must have an opening large enough to allow nail insertion. Some closed-box posterior stabilized femoral component designs may not allow this technique.

As in our case, the literature also describes good results with this technique, with some studies reporting that supracondylar intramedullary nailing seems to be the best treatment for most displaced osteoporotic supracondylar fractures [7, 10].

With this case, supported by basic osteosynthesis principles, the patient was successfully managed with distal femur intramedullary nailing technique, which reinforces this option as a viable treatment alternative, especially for displaced periprosthetic femoral fractures in osteopenic bone.
